# Synthetic Training Enables Deployment on Raw Drone Data: An Attention-Based Framework for Detecting Orphan Wells

**DOI:** 10.3390/s26092573

**Published:** 2026-04-22

**Authors:** Agnese Marcato, Roman Colman, Damien Milazzo, Eric Guiltinan, Zhiwei Ma, Daniel O’Malley, Hari Viswanathan, Javier E. Santos

**Affiliations:** 1 Earth and Environmental Science Division, Los Alamos National Laboratory, Los Alamos, NM 87545, USA; 2 Singapore Institute of Technology, Singapore 138683, Singapore

**Keywords:** drones, machine learning, transformer

## Abstract

Undocumented orphan wells present challenges for subsurface characterization and environmental management due to their unknown locations and varied physical conditions. Magnetic surveys offer a promising pathway for identifying these wells by detecting the magnetic anomalies associated with steel casings. However, magnetometer data are typically high-volume, noisy, and complex, making them difficult to process efficiently with conventional methods. Existing processing methods require heavy preprocessing and achieve unsatisfactory recall scores. In this study, we propose a transformer-based deep learning framework designed to efficiently process hyper-resolute data without extensive downsampling. This is achieved through novel on-the-fly techniques as well as the use of sinusoidal positional encoders to allow the model relative positional awareness. Tests on purely synthetic data show that our model achieves F1-scores of over 90% for line spacings between successive flight paths up to 140 m, enabling surveys to take much sparser flight paths, resulting in more efficient coverage. When applied to real-life data, our model achieves a recall of 70%. This flexible and scalable framework enables the detection of orphan wells from drone data and can be readily adapted to other remote sensing applications.

## 1. Introduction

Undocumented orphan wells (UOWs) complicate ongoing efforts to characterize subsurface infrastructure, allocate remediation resources, and maintain accurate records of legacy energy development. As a result, there is substantial interest in developing reliable methods to locate and document these wells across large survey regions [1,2]. Several detection strategies have been explored, including methane leak detection using dedicated sensors [3,4], satellite-based imaging [5,6], the machine learning-assisted consultation of historical well records [7,8,9], and the identification of magnetic signatures of steel-cased wells using magnetometers [10], the focus of this work.

Despite its promise, magnetometer-based detection presents significant challenges. The high resolution and inherent noise in magnetic data require traditional methods to perform extensive preprocessing or downsampling [11], which substantially reduces data fidelity. For instance, clustering algorithms such as Density-Based Spatial Clustering of Applications with Noise (DBSCAN) [12] and Mean Shift [13] require downsampling to approximately 0.2% of the original resolution, leading to considerable information loss.

More broadly, the efficient processing of large volumes of noisy environmental data remains a well-known research challenge. Conventional unsupervised methods, such as k-means, Mean Shift, DBSCAN, and Gaussian Mixture Models, have been widely applied but struggle to generalize across diverse data modalities and noise patterns. Recent studies have attempted to enhance clustering and detection performance by integrating deep learning approaches [14,15,16,17,18]. Convolutional Neural Networks (CNNs), for example, have shown promise in similar applications; however, they typically rely on heavy interpolation and downsampling to achieve acceptable predictions due to their limited ability to handle sparse data [19]. This preprocessing often introduces artifacts and smooths out high-frequency components, degrading prediction quality [20].

A major bottleneck in applying deep learning to environmental detection is the scarcity of labeled training data. Synthetic data generation offers an effective solution by enabling the creation of large, accurately labeled datasets that mimic physical phenomena [21,22]. In particular, the magnetic dipole equation has been shown to yield realistic synthetic magnetometer signals corresponding to buried anomalies [22,23].

Building on these advances, in this work we introduce a deep learning framework that incorporates a novel positional encoding scheme and leverages physically based synthetic data. We first describe the model and the synthetic data generation process, and then demonstrate that our approach performs effectively in both synthetic and real-world scenarios for orphan well identification. Finally, we discuss how the proposed framework can be extended to a broader range of environmental and remote sensing applications.

## 2. Magnetometer Data

Magnetometer surveys are a key tool for detecting undocumented orphan wells by identifying the magnetic anomalies generated by buried steel-cased wells. In this section, we describe the collection and preprocessing of the magnetometer data used in this study, along with the main challenges associated with its processing and interpretation.

**Figure 1:**
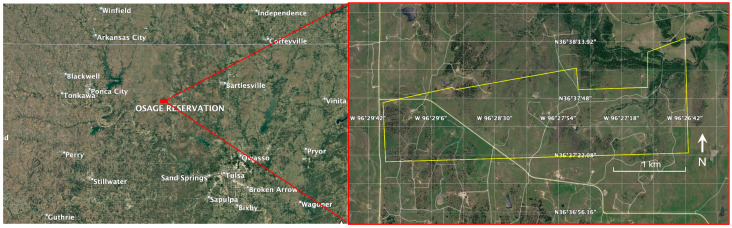
Location of the data collection site.

The data analyzed in this work were collected in the Osage Nation, located in north–central Oklahoma, in Figure 1 the location details are shown.

The magnetometer data used in this study have been acquired using a Sensys R3 magnetometer, which records measurements at a sampling rate of 200 Hz. The sensor is mounted on drones flying at an altitude of approximately 40 m. The drones follow a series of parallel flight paths with an average spacing of 47 m between adjacent lines. We note that flight altitude and inter-line spacing directly influence magnetic anomaly amplitude and spatial sampling density. At higher altitudes, dipole field strength decays rapidly with distance, potentially reducing the detectability of small or weakly magnetized targets. Similarly, wider line spacing may limit the number of measurements capturing the lateral extent of an anomaly. The selected altitude (approximately 40 m) and spacing (approximately 47 m) reflect practical operational constraints related to UAV safety, coverage efficiency, and regulatory considerations. As shown in Section 5, synthetic experiments demonstrate that the model maintains high detection performance even at comparable and substantially larger spacings, suggesting robustness to survey sparsity within realistic operational ranges. To enable efficient data storage and processing, the data are downsampled to a uniform rate of 100 Hz. The use of UAV-mounted magnetometers for detecting shallow metallic targets has been demonstrated in prior studies, including applications to unexploded ordnance detection and lightweight scalar magnetometer platforms for kilometer-scale surveys [24,25]. These works provide evidence that high-resolution aeromagnetic data acquired from low-altitude UAV platforms are suitable for identifying compact ferromagnetic objects, thereby supporting the sensor choice and survey configuration adopted in this study.

**Figure 2:**
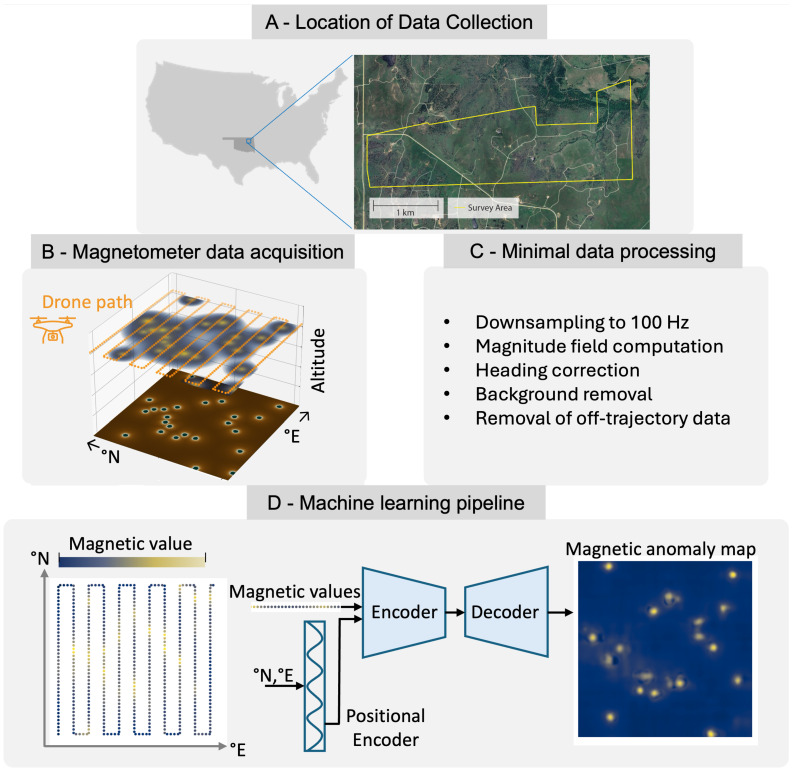
Overview of the workflow used in this study. (**A**) Survey area in northern Oklahoma. (**B**) Acquisition of magnetometer data using drone-mounted sensors. (**C**) Minimal preprocessing of the raw measurements. (**D**) Prediction of candidate orphan well locations using a machine learning model trained on synthetic data.

Standard preprocessing procedures are applied to mitigate systematic errors. Invalid measurements flagged by the sensors are filtered out, and the total magnetic field magnitude,

(1) $$B_{\mathrm{total}}=\sqrt{B_{x}^{2}+B_{y}^{2}+B_{z}^{2}},$$

is computed from the magnetic field components $\left(B_{x},B_{y},B_{z}\right)$, where *B* represents the magnetic flux density. A heading correction is then performed to compensate for the changing alignment of the sensors relative to the Earth’s magnetic field. The Earth’s background field component is subsequently subtracted to isolate the magnetic anomalies associated with subsurface features. Finally, data points located outside the main flight trajectories are removed to retain only relevant survey regions. The data processing workflow can be visualized in Figure 2.

The preprocessing steps described above are limited to standard geophysical corrections (invalid data removal, heading correction, and background field subtraction) and do not involve spatial interpolation, smoothing, or aggressive filtering. The resulting anomaly fields therefore retain the native spatial resolution and noise characteristics of the acquired 100 Hz measurements. Because the machine learning model operates directly on these minimally processed sensor values, the predictive performance reflects the intrinsic quality of the corrected magnetometer data rather than artifacts introduced by heavy preprocessing.

Despite these corrections, magnetometer data remain challenging to process and interpret. The measurements are highly sensitive to a wide range of noise sources, including geological variability, nearby metallic objects, drone orientation and altitude fluctuations, geomagnetic disturbances, and onboard electrical interference [26,27,28]. UAV-mounted magnetometers are particularly susceptible to electromagnetic interference generated by onboard electronics and flight control systems, which can introduce systematic or spectral artifacts into the recorded signal.

Another significant limitation arises from the sparse spatial coverage of real-world surveys. Due to resource constraints, drone flight paths are separated by tens of meters, leaving large unsampled regions where magnetic anomalies may remain undetected. Consequently, reliable interpretation requires the ability to infer well locations from faint and partial edge signals within the data.

Additionally, each drone flight typically lasts several minutes, during which measurements are recorded every 0.04 s resulting in 50,000 to 200,000 readings per flight. This high data density makes processing computationally demanding and often necessitates aggressive downsampling, which leads to loss of resolution and degraded prediction quality. As a result, developing methods capable of handling high-resolution, noisy, and sparsely sampled magnetometer data remains a central challenge in the field.

## 3. Methods

### 3.1. Model and Architecture

Our deep learning framework is based on the Senseiver model [29,30], a transformer-based architecture designed to reconstruct sparse spatial fields. We employ an encoder–decoder structure in which input sensor values and their spatial positions are jointly encoded into a compact latent representation. Cross-attention layers are then used to aggregate contextual information, enabling robust and computationally efficient reconstructions even under conditions of high noise and sparsity.

The model incorporates a spatial encoder that maps positional inputs using sine–cosine positional encodings, allowing the network to infer spatial relationships among sensors. Sensor readings and their positions are concatenated and passed through an attention-based encoder that generates a latent representation. The encoder leverages multi-head cross-attention mechanisms and a query array to summarize inputs into a fixed-size latent space, independent of the number of sensor observations or the spatial domain size. Details of the architecture and the training hyperparameters are reported in Appendix A.

### 3.2. Synthetic Data Generation

Because real-world drone data are limited and well locations are not always documented for survey areas, synthetic data generation plays a crucial role in training and evaluating our model. Wells are randomly positioned within a 256×256 grid, with a minimum center-to-center spacing of 55 units to ensure realistic proximity conditions (reflecting typical well separations of 50–70 m). The magnetic anomalies produced by these wells are computed using the magnetic dipole equation, ensuring physically consistent field generation.

To emulate the sparse coverage typical of drone-based surveys, virtual sensors are placed along evenly spaced vertical flight paths across the grid, mimicking real flight trajectories. This setup provides the model with a realistic framework to learn spatial inference from limited data. Although the current version assumes a constant flight altitude and evenly spaced sensors, these conditions can be varied in future implementations to incorporate more realistic survey complexity.

Synthetic readings are deliberately less dense than real-world measurements to promote generalization to sparse conditions. Variation in well magnetic strengths is introduced according to a distribution derived from observed field data. Additional sources of synthetic noise, such as Gaussian background fields and vertical artifacts, will be incorporated in future iterations to increase realism.

**Figure 3:**
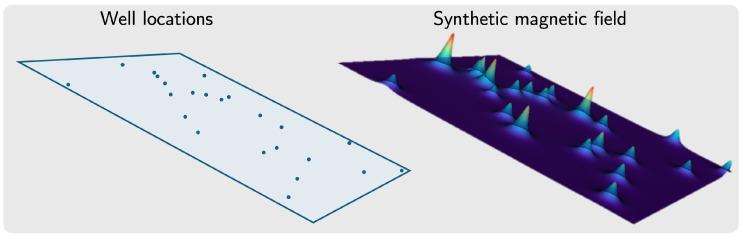
Synthetic sensor values generated using the magnetic dipole equation.

To recreate magnetometer data [23,31,32], we use the magnetic dipole formulation:

(2) $$B\left(r,m\right)=\frac{\mu_{0}}{4\pi }\left[\frac{3(m\cdot r)r}{r^{5}}-\frac{m}{r^{3}}\right],$$

where $\mu_{0}=4\pi \times 10^{-7}$ H/m is the permeability of free space, and r is the position vector from the source to the measurement point. The magnetic moment of a well, $m=(m_{x},m_{y},m_{z})$, is defined as

m=JV,

where *J* is the magnetization constant (dependent on the casing’s magnetic susceptibility) and $V=\pi R^{2}h$ is the well volume, with *R* and *h* representing the well radius and height, respectively. The magnetic moment varies across wells due to differences in these physical parameters. Figure 3 shows an example of data produced, given the location of the wells and their properties the synthetic magnetic field is calculated.

### 3.3. Training on Synthetic Data

For each simulated survey, the model “flies” over the domain according to a specified sparsity level and sampling frequency, recording synthetic sensor values and their positions. These positions, together with their corresponding “floor” coordinates, are mapped through sine–cosine positional encodings to provide spatial awareness. To stabilize training, sensor values are logarithmically scaled.

The encoded sensor values and positional embeddings are passed through the model, which predicts a 2D spatial field, referred to as the predictive space.Model performance is optimized using a mean squared error (MSE) loss between predicted and ground-truth fields. To prevent the model from being overly penalized for small spatial misalignments, we define a 5-unit ε-ball around each ground-truth well location, ensuring that predictions within this tolerance are rewarded. In the 256×256 synthetic grid, this corresponds to approximately 5 m in physical space, given the domain scaling used to match typical well separations of 50–70 m. This tolerance was selected empirically through sensitivity testing to balance localization precision and training stability. Smaller radii led to unstable gradients due to minor positional shifts in predicted centroids, while larger values artificially inflated performance metrics. The chosen value reflects the practical spatial resolution of drone surveys and ensures that predictions remain within a physically meaningful localization error.

### 3.4. Advantages of the Neural Network Framework

Transformer-based neural networks offer a flexible and robust solution to the challenges of magnetometer data processing. They can integrate multiple input modalities, such as magnetometer and methane readings, and maintain predictive accuracy even in noisy or sparsely sampled conditions. Although real methane data are currently limited, incorporating such complementary information illustrates the extendability of the proposed framework.

Neural networks are particularly effective in recognizing faint edge signals, even those representing only 1–10% of a central signal’s strength. By learning both the signal intensity and its spatial variations, the model can triangulate well locations from sparse data, something unattainable for linear models. Moreover, their ability to adapt to varying data density allows the framework to handle high-resolution inputs without excessive downsampling, enabling efficient and accurate large-scale surveys.

## 4. Real Data Deployment

Deploying our model on real-world magnetometer data required several adaptations due to the hyper-resolute nature of the measurements, the presence of noise, and inconsistencies in flight paths. In this section, we outline the procedures developed to handle these challenges, including adjustments to the positional encoding strategy, noise treatment, distribution alignment between synthetic and real data, and flight-path considerations.

The magnetometer data were sampled at 100 Hz, resulting in spatial resolutions several orders of magnitude higher than those used in our synthetic 256×256 grid. Directly processing such dense data would impose prohibitive computational costs. To address this, we adopted an on-the-fly positional encoding strategy in which the position of each sensor is computed individually without reconstructing the entire spatial grid. Since the positional encodings are normalized, the model can process each point independently while preserving spatial consistency. During inference, we scale down the spatial domain to the model’s predictive resolution, pass the corresponding sensor coordinates and values to the network, and subsequently rescale the predictions back to the full domain. This approach allows the use of raw, non-downsampled sensor values while maintaining computational efficiency, resulting in significantly higher-quality predictions than those obtained through conventional downsampling. From a computational perspective, this strategy avoids reconstructing a dense spatial grid prior to inference. A full-grid representation over a domain of size M×M requires $O(M^{2})$ memory to store field values and positional encodings, even if only a subset of locations is observed. In contrast, the on-the-fly encoding processes only the *N* available sensor observations, resulting in memory usage that scales as O(N). In sparse drone surveys, where $N\ll M^{2}$, this distinction becomes substantial. The proposed approach therefore reduces memory requirements and preprocessing overhead by eliminating the need for explicit grid reconstruction.

Noise handling posed a second major challenge. Preliminary tests with additional synthetic noise fields (e.g., Gaussian perturbations) did not improve model robustness, suggesting that the network already exhibits resilience to moderate data variability. However, consistent spikes in sensor readings, likely caused by signal interference during flight, introduced significant artifacts. To mitigate this, we injected controlled Gaussian bumps along simulated flight paths during training, which substantially improved model performance on real-world data.

Matching the statistical distribution of real sensor values was also critical for achieving realistic predictions. Variability in measured well strengths is largely driven by differences in magnetic moment, itself influenced by variations in casing magnetization, geometry, and material properties. To replicate this effect, we scaled the magnetic moment of each synthetic well by a random factor drawn from a two-sided normal distribution whose parameters were estimated from the empirical distribution of real anomaly amplitudes. In particular, the mean and variance of the fitted distribution were chosen to match the first- and second-order statistics of the observed magnetic signal strengths. This adjustment reduces distributional mismatch between synthetic and real data and improves transferability during deployment.

Finally, the stochastic and inconsistent nature of real flight paths introduced additional complexity. To bridge this gap, our training data combined synthetic sensor readings derived from the magnetic dipole model with real flight trajectories extracted from the Osage dataset. The model was trained using 75% of the available flights and evaluated on the remaining 25%, demonstrating strong generalization beyond the training set. In future deployments, we recommend fine-tuning the model on flight patterns that deviate significantly from standard vertical sweeps. As more flight data become available, we expect the model’s generalization capability to extend to virtually any flight configuration due to the strong positional adaptability inherent in its architecture.

### Real-Data Workflow

**Figure 4:**
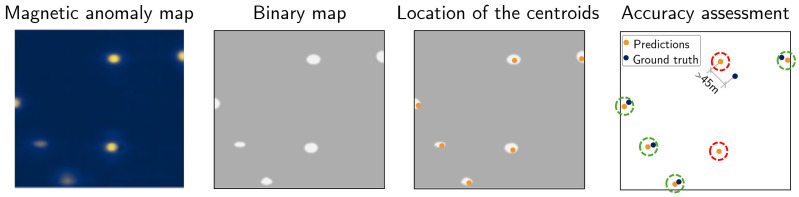
Thresholding process for converting model outputs into well predictions.

In practical terms, the deployment pipeline proceeds as follows: (i) preprocess raw magnetometer measurements to remove invalid or redundant readings; (ii) compute on-the-fly positional encodings for each sensor position; (iii) feed the sensor values and encodings to the model to obtain predictions in the reduced spatial domain; (iv) apply a thresholding procedure to extract likely well locations (Figure 4); and (v) rescale and map the predictions back onto the geographic coordinates of the survey area. This end-to-end workflow enables efficient, high-fidelity inference directly from raw flight data without extensive downsampling or manual preprocessing.

## 5. Results

In this section, we describe the process of deriving predictions from our model outputs and summarize the performance obtained from both synthetic and real-world datasets. Two classes of models were evaluated: (i) *baseline synthetic models*, used to test architecture variants, establish expected performance, and explore potential improvements to flight path configurations, and (ii) *real-data models*, trained using real drone flight paths overlaid with synthetic sensor data, designed to assess the model’s transferability to practical survey conditions.

### 5.1. Prediction Processing

To evaluate performance, we extracted well predictions from the model output using a centroid-based matching procedure. Centroids of connected predicted and ground-truth regions were computed and compared within an ε-radius tolerance. Each match was counted once to ensure uniqueness, enabling the computation of precision, recall, and F1-score as primary evaluation metrics.

### 5.2. Performance on Synthetic Data

**Figure 5:**
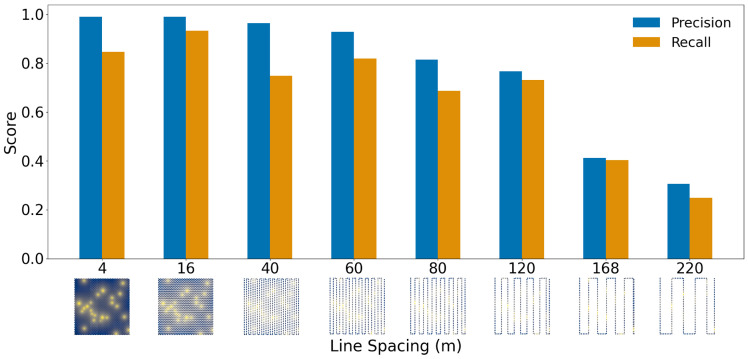
Precision and recall scores for various line spacings (in meters), computed over 500 realizations of well data. Visualization of the line spacings used during evaluation is also shown.

The model demonstrated excellent predictive accuracy on synthetic datasets, showing strong resilience to increased flight sparsity. Even at line spacings of up to 140 m, corresponding to extremely sparse sampling, the network achieved F1-scores exceeding 90%. The best performance was observed for line spacings below 70 m, with F1-scores consistently above 94%. Figure 5 shows the dependence of precision and recall on flight spacing, illustrating the robustness of the framework to sparsity. Given that our real drone surveys operate at an average turnaround spacing of approximately 47 m, these results suggest that future surveys could cover significantly larger areas at reduced cost while maintaining high detection accuracy.

### 5.3. Performance on Real Data

When applied to real-world drone survey data (see Section 4), the model identified 48 potential undocumented orphan wells. Evaluated against the 87 known wells reported in the Bureau of Indian Affairs database [33], the model achieved a recall of 71.2% and a precision of 34%. This corresponds to approximately three candidate locations for every confirmed well. These metrics should, however, be interpreted with caution because the available well inventory for this region is incomplete, which is precisely the motivation for this work. As a result, the reported precision and recall are computed only against the subset of currently documented wells and should therefore be regarded as conservative and illustrative rather than definitive. In particular, some predicted anomalies categorized as false positives may in fact correspond to undocumented or mislabeled wells that are absent from the reference database. The discrepancy between the synthetic and real-world results is expected. The synthetic experiments are conducted in a controlled setting with idealized anomaly characteristics, regular survey geometry, and complete labels, whereas the field data contain substantially greater noise, background variability, positional uncertainty, and incomplete records of well locations. In this sense, the synthetic results establish that the model can learn the relevant magnetic signatures under controlled conditions, while the field deployment evaluates robustness under realistic operational conditions.

**Figure 6:**
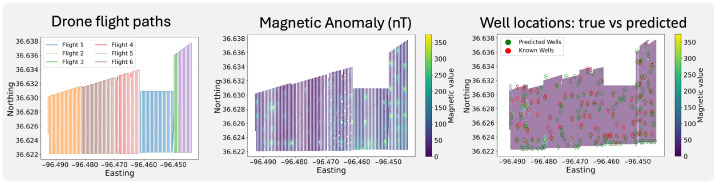
Predicted well locations (blue) versus known wells (red) in measured space, overlaid on a contour plot of sensor values for interpretability.

By calibrating against synthetic model performance at comparable flight spacings, we estimate that the effective recall of the real-data model may be closer to 90%. This value is inferred rather than directly measured, as the available ground-truth database is incomplete and the survey itself was designed to identify undocumented wells. The estimate suggests that a portion of wells either lack a measurable magnetic signature or are misclassified in existing records. Assuming a target precision of 98%, we further infer that roughly 46 of the 48 potential orphan wells detected are likely genuine. Model predictions are shown in Figure 6, illustrating a close correspondence between predicted and known wells.

### 5.4. Comparative Analysis

To benchmark our approach, we compared it against several standard clustering methods commonly applied to magnetometer data, including MeanShift, OPTICS, and DBSCAN. Traditional clustering algorithms struggle with the high noise levels and irregular spatial resolution of real flight data, often requiring heavy interpolation or downsampling that degrades signal fidelity. Table 1 summarizes the comparison, showing that our framework achieves a recall of 0.712, substantially outperforming all baselines. Among classical methods, OPTICS performed best but still lagged behind by more than a factor of two. Because our model is designed to prioritize recall (maximizing identification of potential wells), this metric provides the most meaningful comparison across approaches.

In addition to detection performance, computational considerations further distinguish the proposed framework from classical clustering methods. Algorithms such as DBSCAN, OPTICS, and MeanShift require explicit preprocessing steps, including spatial interpolation, downsampling, or grid reconstruction, to handle the high density and irregular spacing of 100 Hz flight data. These operations introduce additional computational overhead and may generate artifacts that affect clustering outcomes. Moreover, clustering methods are sensitive to hyperparameter choices (e.g., neighborhood radius, minimum cluster size), which often require manual tuning for each survey configuration.

In contrast, our framework operates directly on raw sensor observations without reconstructing a dense spatial grid, and inference scales linearly with the number of measurements provided to the model. While we do not report explicit runtime benchmarks here, the elimination of heavy preprocessing and manual parameter tuning substantially reduces operational complexity in practical deployments.

Overall, these results demonstrate that our transformer-based framework not only generalizes effectively from synthetic to real data, but also surpasses existing clustering methods in both detection accuracy and computational efficiency, even when operating directly on high-resolution 100 Hz magnetometer data without extensive preprocessing.

## 6. Discussion

The results demonstrate that our transformer-based framework provides a powerful and versatile solution for processing magnetometer data in the search for undocumented orphan wells. Compared to conventional clustering-based methods, our model achieves higher recall while requiring minimal data preprocessing or downsampling. Its ability to operate directly on hyper-resolute 100 Hz data offers a major advantage for large-scale survey applications, significantly reducing computational overhead and manual data manipulation.

Beyond orphan well detection, the architecture presented here holds promise for a wide range of remote sensing applications. The model’s capacity to integrate heterogeneous sensor inputs is grounded in its cross-attention mechanism, which aggregates tokenized observations into a shared latent representation independent of sensor modality. Each sensor reading, together with its spatial coordinates, is encoded through the same positional encoding framework, enabling consistent spatial reasoning across modalities. This structure allows additional data streams, such as methane concentration or thermal imaging, to be incorporated without modifying the core architecture. Although real methane data were not available for this study, future multi-sensor deployments could further improve detection performance by combining complementary physical signals.

The model’s demonstrated generalization from synthetic to real flight data underscores the strength of using physically informed synthetic datasets in training. This approach not only addresses the scarcity of labeled data but also provides controlled environments for testing model sensitivity to noise, sparsity, and flight geometry. With additional real-world data and fine-tuning, we expect the model’s recall and precision to improve further, supporting its deployment in large-scale well identification campaigns.

An important practical consideration is the relationship between survey geometry and the minimum detectable magnetic moment of a well casing. For a magnetic dipole, field strength decays approximately as $r^{-3}$ with distance from the source, implying that increased flight altitude reduces anomaly amplitude, while wider line spacing decreases the likelihood of directly sampling the anomaly peak. In our synthetic data generation, magnetic moments were sampled from distributions derived from real survey measurements, ensuring that the training data reflect realistic signal amplitudes. While the present study demonstrates strong detection performance across a range of flight spacings, a formal sensitivity analysis linking minimum detectable magnetic moment to altitude, spacing, and sensor noise floor remains an important direction for future work. Such analysis would further quantify the physically meaningful detectability limits of the proposed framework.

## 7. Conclusions

We have introduced a transformer-based deep learning framework that enables efficient and accurate identification of undocumented orphan wells directly from high-resolution magnetometer data. Our method eliminates the need for extensive preprocessing and downsampling by leveraging on-the-fly positional encodings and a physically grounded data generation strategy. When tested on real flight data, the model achieved superior recall compared to existing clustering algorithms, while maintaining robustness to noise and sparsity.

The proposed framework also offers a scalable foundation for integrating multiple sensing modalities and adapting to new survey conditions. Its architecture naturally accommodates heterogeneous sensor inputs, enabling future incorporation of methane measurements, thermal imagery, or satellite-derived signals within the same modeling framework. By demonstrating strong generalization from synthetic to real data, this work establishes a path toward flexible, field-ready neural networks for environmental sensing. In future work, we aim to incorporate additional datasets, such as methane sensor readings, satellite imagery, and multi-altitude flight surveys, to further improve the model’s robustness and expand its applicability to other geophysical detection tasks.

## Figures and Tables

**Table 1:** Comparison of prediction methods on real magnetometer data. A 45 m matching radius was used for evaluation.

Method	Recall
Our Framework	**0.712**
OPTICS	0.250
MeanShift Clustering Algorithm	0.192
DBSCAN	0.135

## Data Availability

Data will be made available upon request.

## Appendix A. Model Details

The Senseiver model, available at https://github.com/OrchardLANL/Senseiver (accessed on 8 April 2026), was trained for this study. In Table A1 the architecture details and training hyperparameters are summarized.

**Table A1:** Senseiver Hyperparameters.

	Hyperparameter	Value
Training	Learning rate	$1\times 10^{-4}$
	Batch frames	64
	Batch pixels	2048
	Max epochs	292
	Gradient accumulation	1
	Dropout	0.0
Positional Encoding	Type	Fourier features
	Frequency bands	32
	Encoding dimension (2D)	128
Encoder	Latent vectors	64
	Latent channels	16
	Layers	3
	Cross-attention heads	2
	Self-attention heads	2
	Self-attention layers/block	3
Decoder	Latent channels	16
	Cross-attention heads	1
	Output channels	1
